# Low myelinated nerve-fibre density may lead to symptoms associated with nerve entrapment in vibration-induced neuropathy

**DOI:** 10.1186/1745-6673-9-7

**Published:** 2014-03-08

**Authors:** Lars B Dahlin, Helena Sandén, Erik Dahlin, Malin Zimmerman, Niels Thomsen, Anders Björkman

**Affiliations:** 1 Department of Hand Surgery, Lund University, Skåne University Hospital, Malmö, Sweden; 2 Department of Occupational and Environmental Medicine, Sahlgrenska School of Public Health and Community Medicine, University of Gothenburg, Gothenburg, Sweden; 3 Department of Clinical Sciences in Malmö, Hand Surgery, Lund University, Jan Waldenströms gata 5, SE-205 02 Malmö, Sweden

**Keywords:** Vibration exposure, Nerve compression, Carpal tunnel syndrome, Neuropathy, Cerebral plasticity

## Abstract

Prolonged exposure to hand-held vibrating tools may cause a hand-arm vibration syndrome (HAVS), sometimes with individual susceptibility. The neurological symptoms seen in HAVS are similar to symptoms seen in patients with carpal tunnel syndrome (CTS) and there is a strong relationship between CTS and the use of vibrating tools. Vibration exposure to the hand is known to induce demyelination of nerve fibres and to reduce the density of myelinated nerve fibres in the nerve trunks. In view of current knowledge regarding the clinical effects of low nerve-fibre density in patients with neuropathies of varying aetiologies, such as diabetes, and that such a low density may lead to nerve entrapment symptoms, a reduction in myelinated nerve fibres may be a key factor behind the symptoms also seen in patients with HAVS and CTS. Furthermore, a reduced nerve-fibre density may result in a changed afferent signal pattern, resulting in turn in alterations in the brain, further prompting the symptoms seen in patients with HAVS and CTS. We conclude that a low nerve-fibre density lead to symptoms associated with nerve entrapment, such as CTS, in some patients with HAVS.

## Vibration exposure and HAVS

Prolonged exposure to hand-held vibrating tools, which are commonly used in several occupations, may lead to a variety of disorders in the vascular, neural, and musculoskeletal systems. These conditions are collectively known as the hand-arm vibration syndrome (HAVS). Workers exposed to hand-arm vibration may experience tingling and numbness in their fingers and hands and, if such exposure continues, may develop a reduction in perception regarding touch and temperature. Moreover, and crucial from a functional point of view, prolonged exposure eventually leads to impairment of manual dexterity impacting on the patients’ professional life and leisure activities and consequently a lower quality of life [1-3]. Within the Swedish workforce, 15% of employed men and 3% of employed women use hand-held vibrating tools for at least 25% of their workday (Statistics Sweden, 2011) [4]. However, the true prevalence of exposure to hand-held vibrating tools is difficult to assess and depends on the population studied. A recent review concluded that there is substantial evidence of a dose-response relationship between vibration exposure and HAVS [5].

## Nerve compression and HAVS

Among nerve entrapments, carpal tunnel syndrome (CTS) is by far the most common, affecting 3-4% of the general population and with a high rate of surgical intervention [6,7]. The majority of CTS cases are considered to be idiopathic, but there are many known risk factors, such as diabetes, hypothyroidism and vibration exposure. From a clinical point of view, there is an individual vulnerability to peripheral neuropathy and/or CTS following exposure to hand-held vibrating tools. The neurological symptoms of HAVS are similar to the symptoms found in CTS, which makes it difficult to distinguish between the two conditions. Patients with CTS may also experience symptoms from the fingers innervated by the ulnar nerve, which add to the difficulties of making a proper diagnosis [8]. Furthermore, standard clinical testing, such as that for perception of touch, may demonstrate similar results for the two conditions. However, refined diagnostic tests, such as electrophysiology and tactilometry [9-11], allow a distinction to be made between pathology located specifically to the median nerve and/or in the ulnar nerve or a general neuropathy, such as HAVS. Thus, these diagnostic tests may assist in differentiating between CTS and HAVS. A difference in the susceptibility of neuropathy between the median and ulnar nerves has also been detected in some conditions, such as type 2 diabetes [12].

The diagnosis of HAVS, and particularly the peripheral neuropathy seen in HAVS, is largely based on the patient’s own history of symptoms and duration of exposure to hand-held vibrating tools in combination with clinical examination and a variety of sensory tests. There is no single diagnostic test specific for HAVS. Some of the tests used are quantitative sensory tests, e.g. the vibration threshold test and tests for thermal perception and perception threshold with monofilaments [13]. They are all so-called psychophysical tests, since their outcome, which depends on the integrity of the entire somatosensory pathway, is based on an objective physical stimulus that relies on a subjective response from the tested individual. The only objective sensori-neural test to diagnose large-fibre neuropathy is the nerve conduction test, but this measures only the function of the *fastest* conducting myelinated nerve fibres. Thus, in fact, just a limited proportion of the whole nerve fibre population is available for testing. Nonetheless, the electrophysiology test is considered to be the gold standard for the diagnosis of nerve entrapment, such as CTS, as fractionated nerve conduction testing with measurement of conduction velocity and amplitude [14] across the carpal tunnel provides sufficient sensitivity and specificity for the diagnosis. However, the results from the electrophysiology test should always be related to the patient’s symptoms and clinical signs at investigation before a definitive diagnosis is set.

Occupational hand-arm-vibration exposure and forceful repetitive workload are known risk factors for CTS [5,15-20]. A recent meta-analysis shows a strong relationship between a stringent case definition of CTS (symptoms combined with nerve conduction abnormality) and the use of vibratory tools (OR 5.40; 95% CI 3.14 - 9.31), hand force (OR 4.23; 95% CI 1.53, 11.68) and repetitive hand use (OR 2.26; 95% CI 1.73, 2.94) [15]. A better understanding of the factors that may explain individual vulnerability to CTS and neuropathy following exposure to hand-held vibrating tools would provide better treatment possibilities. Furthermore, it would also enhance our ability to advise the patient regarding future exposure to hand-arm vibration. Here, we present current knowledge concerning nerve-fibre density in neuropathies of various origins, focusing on vibration-induced neuropathy, and its relation to symptoms of CTS. Our hypothesis is that low nerve-fibre density may lead to symptoms associated with nerve entrapment. Furthermore, changes in nerve-fibre density may also result in a change in afferent nerve signal pattern, which in turn results in alterations in the central nervous system (CNS). These cerebral changes may further potentiate the symptoms seen in patients with HAVS and CTS. Finally, we will briefly describe techniques that may be of value in the diagnosis of nerve dysfunction in HAVS with and without CTS.

## Nerve compression and neuropathy

### Changes in peripheral nerves due to compression – experimental studies

The pathophysiology of nerve entrapments is complex as it affects all structures in and around the peripheral nerve. The response to compression can be summarized as a combination of disturbance in the intraneural microcirculation, increased vascular permeability, formation of endoneurial oedema, increased endoneurial fluid pressure, demyelination, induction of inflammation, axonal degeneration, fibrosis, new axonal growth, remyelination as well as perineurial and endothelial thickening [21,22]. These responses depend on the nature, magnitude and duration of compression and the coexistence of other diseases, such as diabetes. The most important components in a peripheral nerve trunk, i.e. the axons, respond differently depending on their size. Furthermore, animal experiments have shown that the Schwann cells around the axons also are affected by the compression. Experimental studies have shown that the response is more pronounced in both neurons and Schwann cells in diabetic animals than in healthy ones [23]. The intracellular transport in the axon, i.e. axonal transport, provides the distal part of the nerve with essential substances as well as conveying information from the periphery up to the nerve-cell body. Axonal transport is also more severely inhibited in the sciatic nerve in diabetic rats during compression. Concerning individual nerve fibres, it has been reported from animal studies that large myelinated nerve fibres become demyelinated and degenerate early during nerve compression, while the non-myelinated nerve fibres withstand higher levels of compression and for longer time periods [24-26]. Thus, from a neurobiological standpoint there is supporting evidence that large myelinated nerve fibres are more susceptible to compression than non-myelinated nerve fibres.

### Findings in nerve biopsies from humans

There is limited knowledge concerning the impact of nerve-fibre density as a risk factor in the development of peripheral neuropathy or nerve entrapment symptoms in humans [27]. However, there are indications that a low axonal density may lead to neuropathy [28,29]. To assess nerve-fibre density, a relevant nerve biopsy is required, but potential complications mean that diagnostic nerve biopsies are rarely performed; leading to limited research being done in this field. Recently, we reported that the number of myelinated nerve fibres can be quantified from biopsies of the terminal branch of the posterior interosseus nerve taken from the dorsum of the wrist, at the same level as the median nerve in the carpal tunnel [29]. The risk of complications after such a procedure is low [28-30] in contrast to “classic” sural nerve biopsies [31-33]. In patients with CTS with no other neuropathy, a significant reduction in myelinated nerve fibres and endoneurial capillary densities was found compared to healthy subjects without CTS [29]. More importantly, it was also demonstrated that the decreased myelinated nerve-fibre density was even more accentuated in patients with diabetes and CTS. Thus, a nerve trunk that is not exposed to compression in a diabetic patient demonstrates a reduction in myelinated nerve fibre and endoneurial capillary densities. If these findings from the posterior interosseus nerve are extrapolated to the closely located median nerve, a general hypoxic environment due to reduced vascularity as well as a reduction in nerve-fibre density could predispose to nerve compression symptoms from the median nerve [28].

### Nerve structure and dysfunction

A number of studies have examined the relation between results from nerve biopsies and clinical nerve dysfunction. A significant association between a reduced myelinated nerve-fibre density, clinical signs of neuropathy and electrophysiological dysfunction was demonstrated from sural nerve biopsies in subjects with diabetes, those with impaired glucose tolerance, and those with normal glucose tolerance [34-36]. Furthermore, a correlation was also found between low myelinated nerve-fibre density and progression of nerve dysfunction. These results indicate that a low myelinated nerve-fibre density presents clinically as neuropathy [37,38]. However, the fibre density does not correlate with the glucose-tolerance status, i.e. level of blood glucose, indicating that factors other than the blood glucose level also influence the development of neuropathy. Normal aging affects the nervous system with a progressive reduction in neurons and axons, making the peripheral nerve even more susceptible, probably more so in nerves affected by a concomitant disease, such as diabetes. Therefore, even healthy subjects with already low myelinated nerve-fibre density may be unable to compensate for the normal effect of aging and are thus more prone to develop nerve dysfunction when the nerve is compressed.

## Effects of vibration exposure on nerve fibres

The pathophysiology of vibration-induced neuropathy is complex, but includes morphological changes, such as degeneration of nerve fibres and fibrosis [39,40]. Strömberg et al reported pathological changes in biopsies from the posterior interosseus nerve in patients exposed to vibration, and concluded that demyelination might be the primary lesion in neuropathy following vibration exposure [40]. They also demonstrated that demyelination was followed by oedema, endoneurial fibrosis and, in some cases, even axonal degeneration. This agrees with the findings of Ho and Yu [41] who exposed rabbits to vibration and showed that “destruction of the myelin sheath occurs earlier, more severely, and more frequently than that of the axon”. Similar studies performed on rats [42-44], simulating vibration from hand-held power tools, reported axonal damage and myelin fragmentation in nerves that had been exposed to vibration, explaining the observed increased stress response in the vibration-exposed rat nerves [45].

## Electrophysiological changes in HAVS and CTS

Electrophysiological studies, designed to explain the nature of the nerve injury in HAVS, have provided inconsistent results [9,10,46-48]. Most studies show a reduction in conduction velocity, indicating demyelination, of the *fastest* large myelinated fibres and in later stages, when the patients have more pronounced symptoms, a lower amplitude, indicating a loss of nerve fibres [48]. The abnormalities, independent of clinical symptoms of entrapment, extended from a distally reduced sensory nerve conduction localized to the digits [49,50] to signs of median and ulnar neuropathies proximal to the hand as well as an association with CTS [51-53]. It is important that the electrophysiological examination is done properly [54-56]. Examination of fractionated nerve conduction of the median nerve across the carpal tunnel [14] in vibration-exposed subjects has revealed a bimodal velocity distribution; thus, one group of patients had a significant reduction in conduction velocity, similar to that seen in idiopathic carpal tunnel syndrome, while another group had no signs of nerve compression at the carpal tunnel, suggesting more distal dysfunction at the level of palm or finger, or at the receptor level [9]. Furthermore, distal motor latency at the wrist is an additional variable that can be measured, where it is longer in patients with CTS. Rosen et al [9] also show that patients exposed to vibration have pronounced increased vibration thresholds in the fingers. Taken together, these results imply that a thorough history taken from the patients, as well as that a careful clinical examination and a meticulous electrophysiological examination, also using tactilometry, are essential components in the diagnostic work-up [10,11].

## Outcome of surgery for CTS in HAVS

The outcome of surgery for CTS seems to be less favourable in patients with vibration-induced neuropathy [57-59]. Patients with HAVS may primarily have injuries/alterations in peripheral sensory receptors, large or thin myelinated nerve fibres, and the small-calibre non-myelinated C-fibres with only few patients having additional symptoms of CTS [15]. Thus, a carpal tunnel release operation may not result in symptom relief. As pointed out earlier, the decision to perform surgery on patients with HAVS and a suspicion of CTS requires careful consideration, with adequate preoperative examinations, and the provision of meticulous preoperative information for the patients, similar to the treatment strategies for patients with diabetes and CTS [8].

## Cerebral changes after a nerve injury

Finally, when discussing the complex pathophysiology of neuropathy and CTS in HAVS one should also consider the potential effects on the central nervous system. It is well known that a peripheral nerve injury in the form of a transection or a vibration-induced neuropathy leads to changes in the brain [60,61]. The signal pattern from the injured nerve to the brain is altered, resulting in a reorganisation in the cerebral areas processing sensory information from the hand. The possibilities of interpreting the new signal pattern from the injured nerve are dependent on several factors, where age and higher cognitive functioning are important for the functional outcome. Furthermore, as sensory information is crucial for optimal motor control, a disturbance in afferent nerve impulses from the hand results in disturbed muscle function in the hand. Thus, the mechanisms behind the clinical outcome following HAVS are likely to be found both in the peripheral (PNS) and in the central nervous systems (CNS).

## Conclusion

We conclude that a) hand-arm vibration exposure is associated with an increased risk of CTS; b) hand-arm vibration exposure appears to be associated with diffuse demyelination of nerves in the hand/wrist area and later a reduction in the myelinated nerve fibers and c) these structural changes, seen particularly as a low myelinated nerve-fibre density, may lead to symptoms associated with nerve entrapment, in particular CTS. Thus, a mechanism, based on a low myelinated nerve-fibre density similar to that observed in healthy and diabetic subjects with CTS, may also be present in HAVS. A peripheral neuropathy also results in a cerebral reorganisation. Thus, the clinical symptoms seen in these patients are caused by changes both in the peripheral and central nervous systems. Patients with HAVS and clinical symptoms of CTS should be carefully considered for surgery since the outcome is not always favourable. Patients with HAVS and a suspicion of CTS should be evaluated, through a thorough patient history, careful clinical examination and appropriate blood samples to exclude other causes of neuropathy. Furthermore, a complete electrophysiological examination, including fractionated nerve conduction of the median nerve across the carpal tunnel, and a tactilometry should be performed.

## Abbreviations

CTS: Carpal tunnel syndrome; HAVS: Hand-arm vibration syndrome; CNS: Central nervous system; PNS: Peripheral nervous system.

## Competing interests

The authors declare that they have no competing interests.

## Authors’ contributions

All authors have contributed equally to the production of the manuscript. All authors read and approved the final manuscript.

## Fundings

The research that is referenced from our group is funded by the Research Council (Medicine), Swedish Diabetes Association, Malmö Diabetesförening, Region Skåne, Promobilia, Swedish Society of Medicine, Skåne University Hospital and Lund University.
